# Clinical impact of a metagenomic microbial plasma cell-free DNA next-generation sequencing assay on treatment decisions: a single-center retrospective study

**DOI:** 10.1186/s12879-022-07357-8

**Published:** 2022-04-13

**Authors:** Akira A. Shishido, Myint Noe, Kapil Saharia, Paul Luethy

**Affiliations:** 1 Division of Infectious Diseases, University of Maryland Medical Center, Baltimore, MD USA; 2 Lee Health, Fort Myers, FL USA; 3 Institute of Human Virology, University of Maryland School of Medicine, 22 E Greene St, Baltimore, MD USA; 4 Department of Pathology, University of Maryland School of Medicine, Baltimore, MD USA

**Keywords:** Cell-free DNA, Transplant infectious disease, Next-generation sequencing, Karius©, Sepsis

## Abstract

**Background:**

Metagenomic next-generation sequencing of microbial cell-free DNA (mcfDNA) allows for non-invasive pathogen detection from plasma. However, there is little data describing the optimal role for this assay in real-world clinical decision making.

**Methods:**

We performed a single-center retrospective cohort study of adult patients for whom a mcfDNA (Karius©) test was sent between May 2019 and February 2021. Clinical impact was arbitrated after review and discussion of each case.

**Results:**

A total of 80 patients were included. The most common reason for sending the assay was unknown microbiologic diagnosis (78%), followed by avoiding invasive procedures (14%). The test had a positive impact in 34 (43%), a negative impact in 2 (3%), and uncertain or no impact in 44 (55%). A positive impact was observed in solid organ transplant recipients (SOTR, 71.4%, p = 0.003), sepsis (71.4%, p = 0.003), and those receiving antimicrobial agents for less than 7 days prior to mcfDNA testing (i.e., 61.8%, p = 0.004). Positive impact was driven primarily by de-escalation of antimicrobial therapy.

**Conclusion:**

Clinical impact of mcfDNA testing was highest in SOTR, patients with sepsis and patients who had been on antimicrobial therapy for less than 7 days. Positive impact was driven by de-escalation of antimicrobial therapy which may highlight a potential role for mcfDNA in the realm of stewardship.

**Supplementary Information:**

The online version contains supplementary material available at 10.1186/s12879-022-07357-8.

## Key Points

This is a retrospective study evaluating the clinical impact of mcfDNA testing at a single center. mcfDNA positively impacted clinical care in 43% of cases. Patients admitted with sepsis, patients receiving antibiotics for less than 7 days, and solid organ transplant recipients derived the most benefit from mcfDNA testing.

**Supplementary Information:**

The online version contains supplementary material available at 10.1186/s12879-022-07357-8.

## Introduction

Clinical metagenomic next-generation sequencing (mNGS) is an emerging diagnostic modality that comprehensively analyzes all genetic material in a given sample of fluid or tissue [1]. mNGS platforms sequence millions of small DNA and/or RNA fragments in parallel [1]. Bioinformatic analyses then match sequences to reference genomes for identification [1]. While most conventional molecular diagnostic assays target a single or limited number of pathogens, mNGS of microbial cell-free DNA (mcfDNA) allows for broad-range pathogen detection [1, 2]. The Karius© test (KT; Karius, Redwood City, California) emerged in 2016 making mNGS of mcfDNA widely available. The assay amplifies small fragments of mcfDNA and then matches the sequences to a bank of reference genomes that can reportedly identify 1250 bacteria, viruses, fungi, and parasites [2].

Understandably, a non-invasive assay able to detect multiple pathogens is an attractive prospect for clinicians. However, in comparison to conventional diagnostics, KT is expensive and does not provide antimicrobial susceptibility results. Current data to define the optimal clinical context for the use of the KT assay is limited. The use of KT at our institution has been restricted to tertiary testing in scenarios where conventional assays do not provide a diagnosis or situations that require morbid invasive procedures. mcfDNA sequencing in general has been studied as a complimentary assay in the rapid diagnosis of sepsis, culture-negative endocarditis, pneumonia, invasive fungal infections, brain abscesses and more recently as an adjunct to conventional microbiology cultures in bloodstream infections and prosthetic joint infections [2–8]. Despite results of recent clinical trials and case reports demonstrating the potential value of mcfDNA testing as a diagnostic tool, the overall impact of mcfDNA testing on clinical care is less certain. For example, a multisite retrospective cohort study of adults and children by Hogan and colleagues evaluating the clinical impact of KT found that despite a positivity rate of 61%, KT had a positive clinical impact in only 7.3% of cases [9]. This is in contrast to a single center study evaluating children by Rossoff et al. in which 56% of samples sent for KT provided clinically relevant information [10]. Given the clinical equipoise, we performed a single center, retrospective study to assess the clinical impact of the KT stratified by patient comorbidities, clinical syndromes, days of antimicrobial therapy and indication for testing to identify a context, if any, in which the assay may have the highest clinical impact.

## Methods

### Ethical considerations

The study protocol was approved by the institutional review board (IRB) at University of Maryland, Baltimore (UMB).

### Study design

We performed a retrospective cohort study of adult patients for whom a KT was sent from our institution between May 2019 and February 2021. We predefined clinical impact categories (Table 1) based on criteria used by Hogan et al. and performed comprehensive record reviews for each case [9]. KT at our institution can be requested by Infectious Disease specialists but is only approved by a group of specialists and one of the microbiology laboratory directors who review rationale for testing and perceived impact on the patient’s clinical care. We included and stratified data by patient comorbidities, infectious syndromes, duration of antimicrobial therapy prior to KT testing, reasons for sending the test, and final clinical diagnosis. Cases that fit within multiple predefined categories were included and analyzed in all categories to which they applied. Clinical impact was arbitrated by all authors based on the actions of the treatment team after review and discussion of each case. Any case for which there was not an initial unanimous consensus was decided by a majority vote between the authors. The mcfDNA assay was performed by Karius© as described by Blauwkamp et al. 2019 [2]. Of note, Karius© was not involved in any part of the study other than providing the commercial service of the mcfDNA assay.

**Table 1:** Clinical impact categories and their predefined criteria

Category	Definition
Positive	Test result led to a new diagnosis when conventional tests were negative
	Test result confirmed clinical diagnosis
	Test result led to an earlier diagnosis
	Test result negated invasive or costly procedures or tests
	Test result helped reduce length of hospital stay
	Test result led to the initiation of appropriate antimicrobial therapy
	Test result led to de-escalation or discontinuation of antimicrobial therapies
Negative	Test result led to unnecessary antimicrobial treatment
	Test result led to unnecessary diagnostic investigation or procedures
	Test result led to an unnecessarily prolonged hospital stay
Uncertain or No impact	Test result did not change any clinical management or unable to determine the clinical impact

*Test Result*—result of microbial cell-free DNA assay; *conventional tests*—standard serological, microbiological, and molecular, histopathological and biochemical results

### Statistical analysis

Categorical variables were reported using frequency and percentages. Mean ± standard deviation of age was reported and days of hospitalization reported using median and quartiles. Comparative analysis of categorical variables was conducted by the Fisher’s exact test or Chi-square test as appropriate, and days of hospitalization was compared using Mann–Whitney U test. Statistical tests were performed using SPSS (IBM SPSS Statistics for Windows, Version 27.0. Armonk, NY: IBM Corp) with p-values ≤ 0.05 as the significance threshold.

## Results

A total of 80 patients had KT testing over the course of the study period (Table 2; Additional file 1: Table S1). The median age was 54.5 and 60% of the patients were male. The most common reason for sending the assay was unknown microbiologic diagnosis (78%), followed by avoiding invasive procedures (14%), confirmatory testing (5%) and early diagnosis (3%). Forty-five patients (56%) were immunocompromised (Table 3). The most common immunocompromising condition was hematologic malignancy (27%), followed by solid organ transplantation (26%). Fourteen patients (18%) had prosthetic hardware or grafts, the majority of which were prosthetic heart valves (10%) followed by vascular grafts (7%). The most common clinical syndrome was respiratory failure/pneumonia (31%) followed by sepsis/septic shock (15%). Thirty-seven patients (51%) received more than 7 days of antimicrobial therapy prior to KT testing.

**Table 2:** Patient Demographic and Baseline Characteristics

Total patients	N = 80
Age (years), mean (± SD)	54.3(± 15.4)
Gender, n (%)	
Female	32 (40)
Male	48 (60)
Reason for mcfDNA Assay, n (%)	
Microbiologic diagnosis unknown	74 (78)
Avoid invasive diagnostic procedure	13 (14)
Confirmatory test	5 (5)
Early diagnosis	3 (3)
Types of Clinical Impact, n (%)	
Negative	2 (3)
Positive	34 (43)
Uncertain or No impact	44 (55)
Consistency with final clinical diagnosis, n (%)	
Yes	52 (65)
No	25 (31)
NA	3 (4)

SD—Standard deviation; NA—Not applicable because the KT assay did not meet quality control and did not provide a result; mcfDNA—microbial cell-free DNA; Clinical Impact—defined in Table 1

**Table 3:** Patient characteristics and relationship to clinical impact†

Comorbidities	n (%)	Uncertain or no impact, n (%)	Positive impact, n (%)	P-value	OR	95% CI
Immunocompromised	45 (56)	24 (53.3)	21 (46.7)	0.522		
Organ transplant	21 (26)	6 (28.6)	15 (71.4)	0.003	5.000	1.67–14.95
Stem cell transplant	3 (4)	3 (100)	0			
Solid tumor	2 (2)	2 (100)	0			
Hematologic malignancy	22 (27)	15 (68.2)	7 (31.8)	0.189		
HIV/AIDS	1 (1)	1 (100)	0			
Autoimmune disease	3 (4)	1 (33.3)	2 (66.7)			
Hardware or prosthesis	14 (18)	7 (50)	7 (50)	0.593		
Vascular graft	6 (7)	4 (66.7)	2 (33.3)	0.691		
Prosthetic joint or orthopedic hardware	1 (1)	0	1 (100)			
Mechanical cardiac device	1 (1)	0	1 (100)			
Prosthetic valve	8 (10)	3 (37.5)	5 (62.5)	0.285		
Diabetes	13 (16)	6 (50)	6 (50)	0.626		
Infectious syndrome/clinical diagnosis						
Sepsis/Septic shock	14 (15)	4 (28.6)	10 (71.4)	0.02	4.176	1.176–14.765
Bacteremia	3 (3)	1 (33.3)	2 (66.7)			
Vascular graft infection	7 (7)	4 (57.1)	3 (42.9)	1		
Endocarditis	13 (13)	6 (46.2)	7 (53.8)	0.414		
Respiratory failure/pneumonia	30 (31)	14 (48.3)	15 (51.7)	0.265		
Bone/Joint infection	4 (4)	3 (75)	1 (25)			
CNS infection (meningoencephalitis)	10 (10)	7 (77.8)	2 (22.2)	0.285		
Fever unknown origin	10 (10)	5 (50)	5 (50)	0.74		
Unexplained leukocytosis	2 (2)	1 (50)	1 (50)			
Sinusitis	1 (1)	1 (100)	0			
Skin and soft tissue infection	1 (1)	0	1 (100)			
Others	1 (1)	1 (100)	0			
Antimicrobial agents administered prior to mcfDNA test	72 (90)	39 (55.7)	31 (44.3)	1		
Less than 7 days	35 (49)	13 (38.2)	21 (61.8)	0.004	4.200	1.537–11.476
More than 7 days	37 (51)	26 (72.2)	10 (27.8)	0.004		
No antimicrobial agents prior to mcfDNA test	8 (10)	5 (62.5)	3 (37.5)	1		
Final diagnosis						
Bacterial	26 (31)	15 (60)	10 (40)	0.661		
Fungal	21 (25)	12 (57.1)	9 (42.9)	0.937		
Viral	10 (12)	6 (60)	4 (40)	1		
Non-infectious	28 (33)	14 (51.9)	13 (48.1)	0.555		
Days of Hospitalization before sending mcfDNA test, Median (Q1, Q3)		11.00 (5.00, 21.50)	9.50 (3.00, 18.25)	0.361		

*CI* confidence interval, *CNS* central nervous system, *HIV/AIDS* human immunodeficiency virus/acquired immunodeficiency syndrome, *OR* odds ratio, *Q* Quartile

^†^Some patients had more than one comorbidity and clinical syndrome. 2 patients with negative impact were not included in the analysis

The KT result was consistent with the final diagnosis in 65% of cases and had a positive impact in 34 cases (43%), a negative impact in 2 cases (3%), and uncertain or no impact in 44 cases (55%) (Table 2). The only patient characteristic associated with a positive impact from KT testing was solid organ transplantation (71.4%, p = 0.003). Other variables associated with a positive impact from KT testing in univariate analysis were presence of sepsis (71.4%, p = 0.02) and antibiotic duration less than 7 days prior to mcfDNA testing (61.8%, p = 0.004). No other patient comorbidities, clinical syndromes or variables analyzed yielded a statistically significant association with positive impact from KT testing.

Pathogens were identified via KT in 49/80 cases. Of the pathogens identified 31% were bacteria, 10% were viruses, and 21% were mold/fungi (Table 3). In cases in which pathogens were identified, 55.1% yielded a positive impact (Table 4). In 31/80 cases, KT testing yielded a negative result. In 7 cases, the negative test supported a non-infectious etiology of the patient’s syndrome and antibiotics were either de-escalated or stopped. Positive impact was driven primarily by KT results leading to de-escalation (47%) of antimicrobial therapy (Table 5).

**Table 4:** mcfDNA result type and Relationship to Clinical Impact

Result type	Uncertain, No impact or negative impact, n (%)	Positive impact, n (%)
Positive with quantitative result	19 (45.2%)	23 (54.8%)
Positive with qualitative result	3 (42.9%)	4 (57.1%)
Negative	24 (77.4%)	7 (22.6%)
Did not Meet QC and was not run	3	0

QC—Quality Control, as specified by Karius©

**Table 5:** mcfDNA assay with positive impact, reason for positive impact

Reason for positive Impact	N (%)
Led to new diagnosis	6 (17.6)
Led to confirmation of diagnosis	11 (32.4)
Led to earlier diagnosis	2 (5.9)
Avoided invasive procedure	3 (8.8)
Led to appropriate antimicrobials	6 (17.6)
Led to de-escalation of antimicrobials	16 (47.1)

## Discussion

While mcfDNA testing is an attractive diagnostic modality for its non-invasive broad-range pathogen detection, its clinical impact on clinical decision-making remains poorly defined. In this single-center retrospective cohort study, we show that the overall clinical impact of KT testing for pathogen identification remains low (43%). Additionally, we identified three contextual factors within our cohort wherein a KT test had a positive impact on clinical decision-making: SOTR (71.4%, p = 0.003), sepsis (71.4%, p = 0.02) and antimicrobial therapy for fewer than 7 days prior to assay collection (61.8%, p = 0.004). The clinical impact was driven primarily by mcfDNA testing leading to de-escalation of antimicrobials and confirming clinical diagnosis (Table 5).

Our finding of 43% overall clinical benefit is higher than prior studies evaluating clinical impact of mcfDNA for pathogen detection in CSF (3.4%) [11] and plasma (7.3%) [9]. This may be due in part because KT testing is strictly regulated within our institution. All KT testing is reviewed by an Infectious Disease specialist in consultation with the clinical microbiology director. Therefore, all KT testing is performed on a narrow, vetted patient population. Additionally, 33% of patients in the study by Hogan et al. had a preestablished microbiological diagnosis through conventional testing, whereas in our study KT testing was often performed when diagnosis was in question [9]. Lack of clinical impact was most commonly due to identification of a new organism that was not acted upon or confirmation of a conventional result that was not acted upon [9]. In our analysis, we designated KT confirmation of diagnosis as a positive impact. We based this designation on the fact that in instances of clinical equipoise, additional data supporting a diagnosis may influence management decisions even if no change in treatment ensues. This designation also included cases in which the diagnosis was uncertain but then reinforced by the KT. Although not all would agree that confirmation of diagnosis by an expensive test adds value, we did not assess cost in our analysis and based impact on the reasoning and management decisions of the care teams. We found that confirmation of diagnosis and de-escalation of antimicrobials to be the primary drivers of clinical benefit, accounting for 79.5% of the positively impacting results. Additionally, our results appear consistent with a similarly executed study by Rossoff et al. that explored the diagnostic capabilities of KT testing for pediatric infections [10].

The positive clinical impact observed in our study was largely driven by KT leading to de-escalation of antimicrobials. This benefit was often derived from situations in which the assay either identified pathogens that were felt to not be clinically relevant, or no pathogen at all (Table 5). While different from our study in its scope, a study by Eichenberger et al. demonstrated that mcfDNA persisted in plasma well beyond conventional blood cultures in bloodstream infections and that persistence was associated with an increased risk of metastatic infection [7]. Taken together, the safe de-escalation or discontinuation of antimicrobials in the context of negative or decreasing levels of mcfDNA may highlight a role for mcfDNA in the realm of stewardship or determining antimicrobial course duration.

The fact that the clinical factors associated with positive clinical impact by KT testing were SOTR, sepsis and short antimicrobial courses may be due to both host and environmental factors. Immunocompromised patients who lack adequate T-cell responses may present with atypical presentations of infections less amenable to detection by conventional cultures. Additionally, these patients often receive broad empiric antimicrobial therapy thus reducing the yield of conventional cultures. In theory, KT may have greater impact in these patients given the broader differential of infectious pathogens and lower sensitivity of conventional diagnostics to detect them. This finding appears consistent with Rossoff et al. who found that KT testing netted a higher yield of clinically relevant pathogens in immunocompromised patients (61%) than in immunocompetent patients (35%) [10] as well as prior reports of utility in diagnosing mold infections in immunocompromised patients and opportunistic infections in HIV patients [3, 6, 12–14].

That other immunosuppressed populations in our cohort (i.e., stem cell transplant patients) did not display the same level of benefit from KT testing may be explained by their underrepresentation and small contributing numbers to the overall data as well as standardized algorithmic approach to management. A recent study by Benamu et al. assessed the utility of early KT testing in patients with neutropenic fever by prospectively obtaining mcfDNA within 24 h of fever onset [15]. The authors concluded that KT testing could have allowed earlier optimization of antimicrobials in 47% of patients [13]. While the impact of KT testing in this study was similar to our overall results, the lack of effect we observed specifically in hematologic malignancy and stem cell transplant patients compared to Benamu et al. may be due to several factors. First, real-world management of febrile neutropenia remains institution-specific and protocol-driven. Antimicrobial de-escalation may not occur even in the presence of identified pathogens. Therefore, the lack of observed impact in our study may be because the primary team did not change management based on the KT result even if it identified a true pathogen. Second, the prior study did not base impact on treatment decisions, but instead on an arbitration of whether the KT result *could* have made an impact—thereby attenuating any potential algorithmic impact on clinical management results. Third, Benamu et al. collected KT testing within 24 h of fever onset whereas patients in our study often had KT testing evaluated weeks into their course. Lastly, stem cell patients were underrepresented in our cohort.

There are several possible explanations for why patients with sepsis and those on fewer than 7 days of antimicrobial therapy appeared to benefit from KT testing. First, given that positive impact was driven by antimicrobial de-escalation, patients with sepsis are often on very broad antimicrobial therapy and therefore would be most likely to benefit from data supporting de-escalation. Second, given the severity of illness, septic patients may be less likely to experience de-escalation of antimicrobials in the absence of culture data. Additionally, the overall burden of disease and likelihood of the KT being sent earlier in the clinical course of a septic patient on empiric antibiotics may also contribute to its potential impact. While clinical impact was not directly assessed, the SEP-SEQ trial, a prospective study evaluating diagnostic yield in septic patients did suggest a potential benefit consistent with our results [16].

The KT assay provided uncertain or no impact in 44 cases (55%). Most of these cases were comprised of KT results that yielded no pathogen (77.4%, Table 4). For cases in which the KT yielded at least one organism but did not have a positive impact, the organisms were thought to be commensal or bystander organisms and not true pathogens driving the patient’s clinical picture. This finding highlights that the sensitivity of the KT may affects its specificity when organisms are identified.

In two cases, the KT had a negative impact. In the first case, KT testing suggested HSV-1 (Additional file 1: Table S1). This result prompted the initiation of acyclovir, which was later deemed unnecessary and discontinued after three days. In the other case, the KT result suggested *Streptococcus agalactiae*, while the conventional cultures grew *Streptococcus constellatus*. The discordance led to confusion requiring clarification with Karius© about possible genetic crossreactivty within the assay and the pursuit of additional culture data. While the overall negative impact of KT testing was low, and neither case was particularly detrimental to patient care, these cases highlight the fact that even noninvasive testing is not benign.

The retrospective nature of the study comes with inherent limitations. As a descriptive retrospective study, the data were uncontrolled with a heterogenous patient population and lacked standard comparison to conventional testing. Despite the basis of clinical impact of KT testing on the clinical team’s management, the retrospective arbitration process to assign impact comes with inherent subjectivity. We also were unable to incorporate patient outcomes (i.e., mortality) into the final analysis. Additionally, the retrospective nature of the study does not allow for control of the timing of testing and patient and disease characteristics, therefore there was considerable variability among factors and many underrepresented patient populations and diseases. We also could not account for a benefit based on value as total cost was not collected in our data set. Additionally, the small sample size and retrospective nature allow for only hypothesis-generating conclusions to be made.

In conclusion, while mcfDNA testing is a promising technology for rapid microbial diagnosis, the exact clinical context and impact of the test remain undefined. Our study identifies several factors for which the KT assay may have a higher likelihood of providing clinical benefit: SOTR, sepsis and patients who have received fewer than 7 days of antimicrobial therapy. Positive clinical impact was driven primarily by de-escalation of antimicrobial therapy suggesting a potential role for KT testing in the realm of stewardship. Further studies should explore this relationship and the impact of mcfDNA testing specifically in this context.

## Supplementary Information

**Additional file 1.** **Supplementary Table 1.** Raw daw compiled from retrospective record review. Records were reviewed for 80 patients who had a Karius test sent during the study period. Patient comorbidities, antibiotics courses, conventional microbiological records and clinical notes were reviewed. Clinical utility was determined via arbitration by the study authors.

## Data Availability

De-identified data are available as supplementary material (Additional file 1: Table S1) or by request to the corresponding author.
